# Evolution of Stemless Reverse Shoulder Arthroplasty: Current Indications, Outcomes, and Future Prospects

**DOI:** 10.3390/jcm13133813

**Published:** 2024-06-28

**Authors:** Taku Hatta, Ryosuke Mashiko, Jun Kawakami, Gaku Matsuzawa, Yohei Ogata, Waku Hatta

**Affiliations:** 1 Department of Orthopedic Surgery, Joint Surgery, Sports Clinic Ishinomaki, Ishinomaki 986-0850, Japan; r-mashiko@jss-clinic.com; 2 Department of Orthopaedic Surgery, Tohoku University School of Medicine, Sendai 980-8547, Japan; jun_gene@yahoo.co.jp; 3 Department of Orthopedic Surgery, Iwaki Medical Center, Iwaki 973-8402, Japan; gaku1019@gmail.com; 4 Division of Gastroenterology, Tohoku University School of Medicine, Sendai 980-8574, Japan; gmaps177@gmail.com (Y.O.); waku-style@festa.ocn.ne.jp (W.H.)

**Keywords:** stemless, reverse shoulder arthroplasty, complication, revision surgery

## Abstract

Reverse total shoulder arthroplasty (rTSA) is increasingly being used as a reliable option for various shoulder disorders with deteriorated rotator cuff and glenohumeral joints. The stemless humerus component for shoulder arthroplasties is evolving with theoretical advantages, such as preservation of the humeral bone stock and decreased risk of periprosthetic fractures, as well as clinical research demonstrating less intraoperative blood loss, reduced surgical time, a lower rate of intraoperative fractures, and improved center of rotation restoration. In particular, for anatomical total shoulder arthroplasty (aTSA), the utilization of stemless humeral implants is gaining consensus in younger patients. The current systematic review of 14 clinical studies (637 shoulders) demonstrated the clinical outcomes of stemless rTSA. Regarding shoulder function, the mean Constant-Murley Score (CS) improved from 28.3 preoperatively to 62.8 postoperatively. The pooled overall complication and revision rates were 14.3% and 6.3%, respectively. In addition, recent studies have shown satisfactory outcomes with stemless rTSA relative to stemmed rTSA. Therefore, shoulder surgeons may consider adopting stemless rTSA, especially in patients with sufficient bone quality. However, further long-term studies comparing survivorship between stemless and stemmed rTSA are required to determine the gold standard for selecting stemless rTSA.

## 1. Introduction

Reverse total shoulder arthroplasty (rTSA) is increasingly being used as a reliable surgical option for rotator-cuff-deficient glenohumeral arthropathies, glenoid deformity due to primary osteoarthritis, proximal humerus fractures, rheumatoid arthritis, chronic glenohumeral dislocation, and failed hemiarthroplasty (HA) or anatomic total shoulder arthroplasty (aTSA) [1]. The clinical application of rTSA has been adopted for decades, with the first theoretical design developed in 1972 [2]. The primary concept was to reconstruct a stable medialized and distalized center of rotation that can achieve long-term survival of the glenoid components with decreased shear stress at the implant–glenoid interface [3]. The current objective of the rTSA is to create a functional shoulder, especially in cases with deteriorated rotator cuff structures. From 2000 to 2010, the nationally adjusted population rate of shoulder arthroplasties increased 5.0-fold in the United States [4]. The efficacy of rTSA has recently been recognized, with reliable pain relief and excellent functional outcomes. However, there are concerns regarding long-term survivorship and the occurrence of rTSA-related complications. In particular, the long-term outcomes of rTSA in specific populations, such as younger, more active, more obese, and/or more porotic populations, require further investigation [5,6,7].

Traditionally, the humeral component for shoulder arthroplasties has been composed of a long stem that has been utilized to stabilize the humerus. Although a short stem has recently been adopted in some prostheses, the majority of clinical studies assessing the usefulness of rTSA have investigated the outcomes of long-stemmed implants [8,9,10]. The advantages of stemmed implants include augmentation with cement, particularly in patients with poor bone quality [11]. While the survivorship of stemmed rTSAs has been shown to be satisfactory at 10–15 years, cases that require revision surgery are still common [8,10,12,13,14,15,16].

Stemless humeral components for aTSA are being increasingly utilized in the United States and have been compared with traditional stemmed implants in radiographic and patient-reported outcome measure (PROM)-based studies. According to clinical reports, stemless shoulder arthroplasties have similar implant longevity and PROMs to stemmed arthroplasties [17,18,19,20,21,22]. Recent studies have demonstrated that stemless implants have been shown to result in less intraoperative blood loss [23], reduced surgical time [23,24], a lower rate of intraoperative fractures [25], and improved center of rotation restoration [26].

On the other hand, recent biomechanical and radiological studies indicated a potent influence of the technical gap for implantation on the durability of stemless rTSA. Of these, the neck–shaft angle at the osteotomy of the proximal humerus may affect the primary fixation of stemless rTSA [27]. Using specific surgical devices, however, surgeons should note the deviation in performing osteotomy or implantation in terms of resection height, inclination, or retroversion angle [28].

Whether stemless rTSA could become equivalent to or even superior to the gold standard of stemmed rTSA remains controversial [29]. Future studies should focus on specific populations in which stemless rTSA should be used instead of stemmed rTSA. This review aimed to discuss the evolution, current indications, and reported outcomes of stemless rTSAs, especially with a focus on the pooled incidence of overall complications and revision surgery following stemless rTSA.

## 2. Methods

### 2.1. Literature Search and Data Extraction

**Figure 1:**
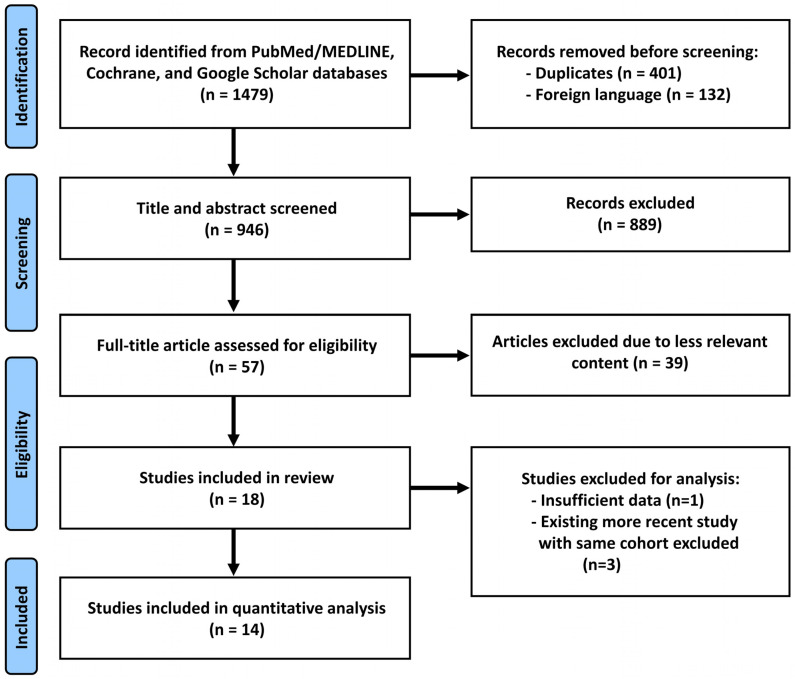
PRISMA flowchart [30,31,32].

A systematic review of the clinical application of stemless rTSA in patients with shoulder disorders was conducted. The MEDLINE/PubMed, Cochrane, and Google Scholar databases were searched in April 2024, according to our methodology, which adhered to the PRISMA guidelines for identifying and evaluating relevant studies (Figure 1). The search strategy employed a combination of the following keywords: (stemless OR non-stemmed) AND (reverse) AND (shoulder arthroplasty OR shoulder replacement). Additional articles were identified by examining the reference lists of articles selected for full-text review. Two independent researchers (T.H. and R.M.) determined that each article was eligible for inclusion by carefully reviewing its contents. In the case of disagreement, a third researcher (J.K.) was consulted to reach a consensus. Finally, 18 studies were included in this review to identify the clinical and radiologic outcomes of stemless rTSA. Furthermore, 14 studies were included for a quantitative analysis to investigate functional improvement, as well as complication and revision rates.

However, the total number of articles reporting outcomes in patients who underwent stemless rTSA was insufficient for some focus in this review. Therefore, relevant articles, including data from stemless aTSA, were also utilized after consensus among the three researchers was obtained.

### 2.2. Quality Assessment

Two independent researchers (T.H. and R.M.) assessed the risk of bias using the Joanna Briggs Institute (JBI) Critical Appraisal Tools for use in JBI Systematic Reviews for prevalence studies [33]. The risk of bias was categorized as low (≥70%), moderate (50–69%), and high (≤49%), based on the percentage of “yes” responses to 9 questions. All discrepancies were discussed and resolved by consensus in consultation with a third researcher (J.K.).

### 2.3. Statistical Analyses

Clinical studies demonstrating the incidence of complications and revision surgery were included in the current meta-analysis. The Freeman–Tukey double arcsine method and inverse variance method were utilized for calculating the pooled proportion and corresponding 95% confidence interval (CI) of prevalence. A random-effects model was used to pool the data, and statistical heterogeneity among studies was evaluated using Cochran’s Q test (significant at *p* < 0.10) with quantifying via the I2 statistics [34]. I2 statistics values were categorized as low (<30%), moderate (30–59%), substantial (60–75%), and considerable (>75%) heterogeneity. Publication bias was assessed qualitatively using funnel plots and quantitatively using Egger’s test (significant at *p* ≤ 0.10) [35]. Statistical analyses were conducted using R version 4.2.1 (R Foundation).

## 3. Evolution of Stemless rTSA

The first design for the “canal-sparing” stemless shoulder arthroplasty system was developed in 2003, but it did not draw much attention until the middle of 2010 when stemless implants received attention based on the following theoretical advantages: preservation of humeral bone stock, reduced periprosthetic fracture risk, higher adaptability during implantation, easier implantation in cases of altered anatomy such as post-traumatic malunion, and less complex revision surgery in case of failure of the stemless device [4,36,37,38]. Over the decades, alterations in implant material, design, and geometry, in addition to supporting evidence, have been carried out to improve the overall function and long-term durability of stemless shoulder prostheses.

### 3.1. Implant Design

The design of stemless humeral prostheses has also changed. The initial model of the Total Evolution Shoulder System (TESS; Zimmer Biomet, Warsaw, IN, USA) was introduced in 2003 as a first-generation stemless shoulder prosthesis. This is composed of six-branched anchors like a “corolla,” which enable it to impact into the humeral metaphysis. However, with this configuration, there is a risk of contact with the lateral cortex of the humerus during impaction, which may result in intraoperative fractures. The structure of the implant assembly between the anchor and the surface portion is disadvantageous, causing metallosis [39]. The modified TESS model was subsequently developed in 2005, and the stemless rTSA system included a one-piece cup-configured corolla to increase the contact area of the humerus [39]. Kadum et al. (2011) first reported the clinical outcomes of stemless TESS prostheses in 17 rTSA patients, with short-term follow-up (range 9–24 months). Although specific outcomes specific to TESS rTSA were not described, complications included instability secondary to malpositioned glenosphere in one patient and dislocation secondary to migrated humeral component in one patient [30]. A clinical study by Ballas and Beguin (2013) focused on the outcomes of stemless TESS rTSA and demonstrated an implant revision rate of 7.1% (4 of 56 shoulders) with an average follow-up period of 4.8 years. Among these, there were no cases of periprosthetic humeral radiolucency, migration, or loosening of the stemless component. Functionally, the mean Constant-Murley Score (CS) improved from 28 (standard deviation (SD): 8) preoperatively to 62 (SD: 12) postoperatively [40]. There have been five additional studies with a specific focus on stemless TESS rTSA; at an average of 17.5–101.6 months of follow-up of 189 shoulders in 184 patients, the implant survival rate of stemless TESS rTSA was reported to reach 94.7%. Notably, no patient was found to have humeral-implant-associated complications in the modified corolla [23,41,42,43,44].

As another stemless metaphyseal rTSA implant released around the same period, the Vesro Shoulder System (Innovative Design Orthopedics, Theale, UK) has been clinically utilized since 2005. It is composed of a short, non-stemmed metaphyseal implant with three tapered fins to achieve immediate metaphyseal press-fit stabilization. Atoun et al. (2014) first reported the clinical outcomes of 31 patients who underwent Verso rTSA with an average follow-up of 3 years. The mean CS improved from 12.7 preoperatively to 56.2 postoperatively. Regarding postoperative complications, one patient had an acromion stress fracture, and five patients sustained traumatic periprosthetic fractures after falls [31]. Several clinical studies have also reported satisfactory outcomes, with a 6.1–13.0% revision rate at an average of 36–50 months of follow-up [32,45]. Most recently, Virani et al. (2021) reported the clinical outcomes of 22 shoulders in 21 patients who underwent Verso rTSA with an average follow-up period of 78 months (range 60–114 months). The mean CS improved from 18 preoperatively to 72 postoperatively, and postoperative complications included a 0 periprosthetic infection in one patient and periprosthetic fractures after falls in two patients [46].

With the prospect of promoting minimally invasive TSA, several companies have commercialized stemless rTSA as next-generation implants. EasyTech (FX Solutions, Viriat, France) was developed in 2011 and has an impacted cage with peripheral serrated pegs. The onlay component was originally used to demonstrate that the entire concave articular part is located above the resection plane. A clinical study demonstrated an implant revision rate of 7.0% (8/115 cases) after 24 months of follow-up. Regarding stemless-implant-associated complications, five patients showed humeral loosening. Functionally, the mean CS improved from 32.5 (SD: 10.3) preoperatively to 61.8 (SD: 15.6) at the 24-month follow-up [47]. The Comprehensive Nano Reverse system (Zimmer Biomet) was developed in 2012 and has an onlay tray system with six thick and short fins. However, for rTSA, this onlay tray system turned out to be insufficient for fixation, with a potential risk of varus seesaw motion; consequently, this system was withdrawn from the market. To date, a clinical study has reported the outcomes of Nano rTSA in 15 patients, with an average follow-up of 27 months (range 9–24 months). Functional improvement was obtained, with the mean CS ranging from 30 (SD: 18) preoperatively to 60 (SD: 18) postoperatively. However, 4 of 15 cases required revision surgeries, including postoperative migration of the humeral component in two patients, instability secondary to an episode of cerebrovascular stroke in one patient, and traumatic periprosthetic fracture in one patient [48].

In 2015, SMR Reverse (Lima Corporate, Villanova, Italy) was designed using a proximal peripheral ring and three fins. This inlay system uses a polyethylene (PE) glenosphere and metal humeral insert to prevent the occurrence of PE wear debris. Schoch et al. (2021) reported the clinical outcomes of 52 patients who underwent stemless SMR rTSA, with a minimal follow-up of 24 months (range 24–41 months). Functionally, the mean CS improved from 24.9 (SD: 9.8) preoperatively to 72.4 (SD: 8.7) at 2 years of follow-up. Radiologically, gross loosening of the humeral component was observed in one case. Postoperative complications included deep infection in one patient and periprosthetic fracture in one patient who required revision surgery [49]. To date, there have been three studies with a specific focus on stemless SMR rTSA. In 111 shoulders in 110 patients, the revision-free rate of stemless SMR rTSA was reported to reach 96.4% with an average follow-up period of 35.8 months. Radiologically, a radiolucent line around the stemless implant was observed in 25 of 107 shoulders (23.4%). Nevertheless, it was notable that no implant-associated complications requiring revision were observed in patients with SMR rTSA [49,50,51].

### 3.2. Implant Material

Currently available humerus components are made of cobalt–chrome or titanium alloy with a full hydroxyapatite coating and a titanium plasma spray.

Hydroxyapatite coating was adopted to improve fixation strength, with the initial introduction to hip and knee arthroplasties in the 1980s [52]. This coating has been shown to accelerate bone ingrowth via its inherent osteoconductive properties [53], with a number of studies supporting minimized micromotion at the implant–bone interface [54,55,56].

Porous plasma spray, which is adopted in most cementless arthroplasties, has been recognized as a major evolution. A randomized controlled study clarified the efficacy of these properties in preventing subsidence in patients who underwent total knee arthroplasty [57].

### 3.3. Biomechanical Properties

Traditionally, the presence of a stem component has been considered essential for achieving rigid stability in the humeral canal. However, biomechanical advancement may enable sufficient fixation strength within the humeral metaphysis.

Ryan et al. (2023) biomechanically investigated the differences in initial strength against torsional load among long-, short-, and stemless implants [58]. They found that the force required to cause failure in synthetic humerus implanted with stemless components was significantly decreased compared with those with long or short stems. They emphasized the importance of metaphyseal cancellous bone quality for stable fixation with stemless implants. Cunningham et al. (2024) investigated the effect of the neck–shaft angle on the primary fixation of stemless rTSA using finite element analysis. They found that lower neck–shaft angles with more varus placement may increase bone-implant distraction in simulated activities of daily living. Therefore, appropriate osteotomy of the humeral head with a higher neck–shaft angle and cancellous bone quality can be considered important for achieving stable fixation in stemless rTSA [27].

## 4. Indication Considerations

With the advancement of modern design, the findings of several studies have encouraged surgeons to use stemless shoulder prostheses. In particular, the stemless shoulder prosthesis may have advantages in younger patients since bone stock preservation is preferred. However, the use of stemless shoulder prostheses in patients with decreased bone quality due to conditions such as osteoporosis or rheumatoid arthritis remains a concern. However, for such patients, there might be a great need for a less invasive stemless shoulder prosthesis.

### 4.1. Young Age

Colasanti et al. (2023) investigated the treatment options for which consensus was gained among international shoulder surgeons for the treatment of osteoarthritis in shoulders under 50 years of age [59]. Notably, 79% of surgeons selected stemless humeral components for HA or aTSA as the proposed surgical procedure. Recent studies have demonstrated that a growing number of young patients are required to undergo rTSA. A clinical investigation utilizing the New Zealand Joint Registry records reported that younger patients undergoing reverse shoulder arthroplasty demonstrated higher implant retention rates than older patients [5]. Moreover, the Australian Orthopaedic Association National Joint Replacement Registry demonstrated that among patients aged <55 years, the revision rate did not differ between aTSA and rTSA. In contrast, patients aged 55–64 years of age had a relatively low revision rate after rTSA [60]. Regarding other risk factors, younger age at the time of surgery may be associated with the periprosthetic infection rate [61].

### 4.2. Obesity

The potential impact of obesity on patients who require TSA should be noted. In contrast to hip or knee arthroplasties, in which increased weight-dependent micromotion could be present, shoulder arthroplasty may have less impact on the occurrence of complications in obese patients. Hatta et al. (2017) demonstrated that increased body mass index (BMI) had no significant impact on postoperative complications in patients who underwent shoulder arthroplasty [61]. In contrast, Gruson et al. (2023) reported that morbid obesity (BMI > 40 kg/m^2^) may not be associated with increased operative time, intraoperative total blood volume loss, or perioperative medical or surgical complications after aTSA. However, it was predictive of increased length of hospital stay [62]. Moreover, Theodoulou et al. (2019) reported greater risks of dislocation, fracture, and revision with increasing BMI in shoulder arthroplasties [63]. Regarding the surgical procedure, Phillips et al. (2023) investigated the effect of morbid obesity on the healing rates of lesser tuberosity osteotomy for stemless and stemmed aTSA [64]. Although there were no differences in the healing rates between stemless and stemmed aTSA, significantly increased BMI was found in patients with a lack of healing compared with those with a healed lesser tuberosity, with a failure rate of 9.4% for BMI 30–40, 12.5% for BMI 40–50, and 28.6% for BMI > 50. Although lesser tuberosity osteotomy is less common in patients who undergo rTSA, morbid obesity is a potential risk factor for nonunion when lesser tuberosity osteotomy is performed.

### 4.3. Osteoporosis

Osteoporosis is frequently observed in patients who undergo aTSA or rTSA. Although there has been a lack of detailed investigation, regional osteopenia was noted to be a relative contraindication to stemless shoulder prostheses due to an association with early humeral component loosening [65,66]. Primary and secondary stability are important factors in implant stability, and patients with osteoporosis may have concerns regarding both. For primary stability, surgeons must carefully assess bone quality at the humeral metaphysis, in which the stemless TSA is embedded. Although preoperative assessment tools, including plain radiography, CT, BMD, and/or MRI, are examined, no objective tests are available to determine the bone quality of a stemless prosthesis [67,68]. For secondary stability, stemless shoulder implants mainly rely on osseointegration for rigid fixation of the metaphyseal components. The quality of the bone for this response has been a concern in patients with osteoporosis or osteopenia. A biomechanical study investigated the micromotion of a single stemless humerus component using finite element analysis and demonstrated that increased micromotion was significantly dependent on decreased cancellous bone density [69,70]. Accordingly, surgeons should note the importance of preoperative or intraoperative assessments to ensure adequate bone quality when selecting a stemless implant.

## 5. Outcomes from Quantitative Analysis

A total of 14 clinical studies (637 shoulders in 629 patients) were included in the quantitative analysis to clarify the current outcomes of stemless rTSA [23,40,41,42,43,44,45,46,47,48,49,50,51,71]. The analysis included six TESS studies (272 shoulders of 266 patients), three Verso studies (127 shoulders of 126 patients), three SMR studies (108 shoulders of 107 patients), one EasyTech study (115 shoulders of 115 patients), and one Nano study (15 shoulders of 15 patients). The mean age at the time of surgery was 71.1 years, and the mean follow-up period after surgery was 40.6 months (Table 1).

Regarding PROMs to assess shoulder function, CS was the most frequently used among the studies: 10 studies for preoperative assessment and 11 studies for postoperative assessment. According to quantitative analysis, the mean CS in patients who underwent stemless rTSA improved from 28.3 preoperatively to 62.8 postoperatively (Table 2).

### Incidence of Complications and Revision Surgery from Meta-Analysis

Among 637 shoulders from 14 studies, complications were reported in 82 cases, with the incidence ranging from 0% [71] to 34.6% [50]. Regarding the quality assessment of 14 studies based on the JBI Critical Appraisal Tools, 10 studies were categorized as being at low risk, and 4 studies were at moderate risk (Supplementary Table S1).

**Figure 2:**
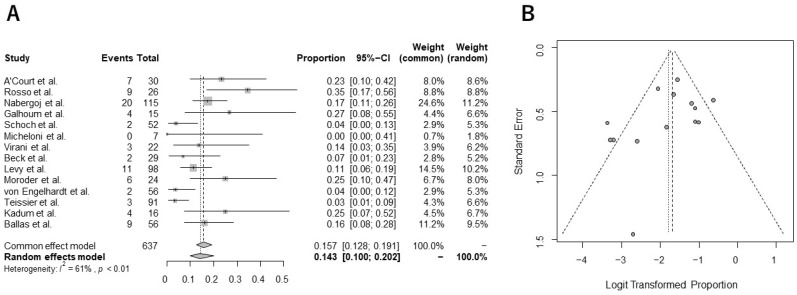
Forest plot (**A**) and funnel plot (**B**) in the analysis of the overall complication rate following stemless rTSA [23,40,41,42,43,44,45,46,47,48,49,50,51,71].

**Figure 3:**
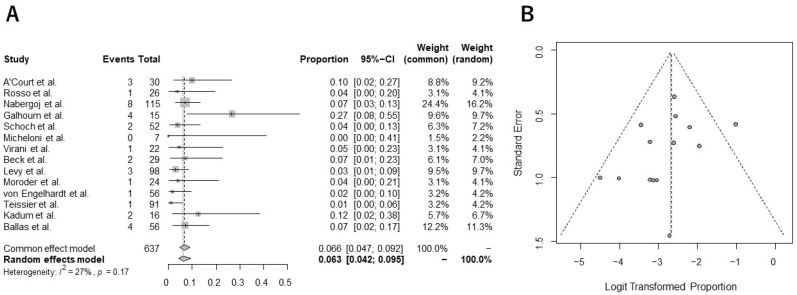
Forest plot (**A**) and funnel plot (**B**) in the analysis of the revision surgery rate following stemless rTSA [23,40,41,42,43,44,45,46,47,48,49,50,51,71].

The pooled incidence of overall complications following stemless rTSA was 14.3% (95% CI, 9.9%–20.2%), with substantial heterogeneity across studies (I^2^ = 61%) (Figure 2A). Common postoperative complications included instability/displacement (n = 16), periprosthetic fracture at the humerus (n = 12), and displacement/malpositioning/migration of the humeral implant (n = 11, Table 3). The pooled incidence of revision surgery after stemless rTSA was 6.3% (95% CI, 4.1%–9.5%), with low heterogeneity (I^2^ = 27%) (Figure 3A). No potential publication bias for the incidence of overall complications or revision surgery was confirmed by Egger’s test (*p* = 0.19, 0.20, respectively). The funnel plots are shown in Figure 2B and Figure 3B.

## 6. Comparative Analysis of Stemmed and Stemless rTSA

With recently reported outcomes, it has been recognized that both stemmed and stemless shoulder arthroplasties achieve satisfactory outcomes in terms of pain relief, functional recovery, and implant survivorship. Recently, the focus has been on comparing these implant designs. As seen in Table 4 and Table 5, clinical studies comparing stemmed and stemless shoulder arthroplasties demonstrated that these implant types showed no significant association with any patient characteristic.

### 6.1. Clinical Outcomes

Three studies compared the short-term clinical and radiological outcomes between stemmed and stemless rTSA [23,42,51]. In a study with approximately 3 years of follow-up, Moroder et al. (2016) showed no differences in the postoperative Constant Score, American Shoulder and Elbow Surgeons (ASES) score, muscle strength, and range of shoulder motions. Radiologically, nine cases (38%) of stemmed and two cases (26%) of stemless rTSA demonstrated grade 1 or 2 scapular notching [23]. More recently, A’Court et al. (2024) compared stemmed and stemless rTSA with a minimal follow-up of 2 years. There were no differences in the postoperative range of shoulder motion, Oxford Shoulder Score, or ASES score. Radiologically, the stemless group included osteolysis around the greater tuberosity in three cases (10%) and periprosthetic radiolucent lines in six cases (20%) [51].

However, several studies evaluated outcomes in comparison with stemmed aTSA. Looney et al. (2022) performed a systematic review and meta-analysis comparing the outcomes of stemmed and stemless aTSA [75]. They utilized four randomized controlled trials, including 229 stemmed and 358 stemless aTSA cases [22,72,73,74]. They demonstrated no differences in the postoperative forward flexion, abduction, or external rotation. Moreover, no differences were found between the stemmed and stemless designs in the occurrence of humeral fractures or the risk of revision surgeries. Further comparative studies on long-term outcomes are required to validate the selection of stemless rTSAs.

### 6.2. Complications

Complications after rTSA have been reported. Several investigations have attempted to reduce the incidence of complications using advanced biomechanical and kinematic approaches [76,77]. However, knowledge of complications after stemless rTSA is insufficient. Moroder et al. (2016) investigated the incidence of postoperative complications after stemmed and stemless rTSA [23]. Four cases (17%) of stemmed rTSA and six cases (25%) of stemless rTSA had complications; however, stemless-component-related complications were not observed. A’Court et al. also reported three cases (10%) of stemmed and stemless rTSA in which revision surgery was required [51].

The incidence of humeral periprosthetic fractures has been reported to be lower in stemless prostheses (0.89%) than in stemmed prostheses (1.6–19.4%) [78,79]. A multicenter study analyzed eight patients (four rTSA and four aTSA) who had sustained a postoperative periprosthetic fracture following stemless shoulder arthroplasty and reported that conservative treatment seemed to be appropriate in patients with low or non-displaced fractures without implant loosening. One of the four stemless rTSA patients had fibrous nonunion at the greater tuberosity. However, the functional outcomes after conservative treatment were maintained in all patients, and no implant loosening was observed [80].

## 7. Conclusions

As designs and techniques have improved over time, stemless rTSAs are evolving and gaining popularity, with recent studies showing satisfactory outcomes. The current review has demonstrated the evolution of and currently available evidence for stemless rTSA and indicates that shoulder surgeons may consider adopting stemless rTSA, especially for patients with sufficient bone quality:

- The current meta-analysis demonstrated that the pooled overall complication and revision rates were 14.3% and 6.3%, respectively;
- Comparative studies may indicate equivalent functional recovery and incidence of complications between stemmed and stemless prostheses;
- Further long-term studies comparing the survivorship between stemless and stemmed rTSAs are required to determine the gold standard for selecting stemless rTSA.

## Figures and Tables

**Table 1:** Patients’ characteristics in clinical studies with stemless rTSA.

Study	Year	Mean Age Year (SD)	Sex (Male %)	Final Number of rTSA in Analysis	Implant	Mean Follow-Up Months (SD)
A’Court et al. [51]	2024	64.3 (11.4)	40	30	SMR	37.5 (14.0)
Rosso et al. [50]	2024	70.1	54	26	SMR	46.8
Nabergoj et al. [47]	2023	68.7	47	115	EasyTech	24
Galhoum et al. [48]	2022	70 (7)	NS	15	Nano	27 (6)
Schoch et al. [49]	2021	61.2	62	52	SMR	29.3
Micheloni et al. [71]	2019	73.1 (8.0)	29	7	Verso	6.4 (1.3)
Virani et al. [46]	2021	76	NS	22	Verso	78
Beck et al. [41]	2019	72.4 (6.7)	19	29	TESS	101.6
Levy et al. [45]	2016	74.4	20	98	Verso	50
Moroder et al. [23]	2016	75.6 (4.6)	29	24	TESS	35.2 (14.6)
von Engelhardt et al. [44]	2015	73.2 (7.8)	NS	56	TESS	17.5 (10.2)
Teissier et al. [43]	2015	73	70	91	TESS	41
Kadum et al. [42]	2014	69	63	16	TESS	35
Ballas et al. [40]	2013	74	29	56	TESS	56

SMR (Lima Corporates), EasyTech (FX Solutions), Nano (Zimmer Biomet), Verso (Innovative Design Orthopedics), TESS (Zimmer Biomet). rTSA: reverse total shoulder arthroplasty, SD: standard deviation. NS: not stratified.

**Table 2:** Functional improvement and complications in clinical studies with stemless rTSA.

Study	Year	Preop. CS (Mean, SD)	Postop. CS (Mean, SD)	Incidence of Complications (%, n)	Incidence of Revision (%, n)
A’Court et al. [51]	2024	NR	NR	23.3 (7)	9.0 (3)
Rosso et al. [50]	2024	44.1 (18.7)	83.1 (10.1)	34.6 (9)	3.8 (1)
Nabergoj et al. [47]	2023	32.5 (10.3)	61.8 (15.6)	17.4 (20)	7.0 (8)
Galhoum et al. [48]	2022	30 (18)	60 (18)	26.7 (4)	26.7 (4)
Schoch et al. [49]	2021	34.9 (9.8)	72.4 (8.7)	3.8 (2)	3.8 (2)
Micheloni et al. [71]	2019	21.6	56.9	0 (0)	0 (0)
Virani et al. [46]	2021	18	72	13.6 (3)	4.5 (1)
Beck et al. [41]	2019	13	60.5	6.9 (2)	6.9 (2)
Levy et al. [45]	2016	14	59	12.2 (12)	3.1 (3)
Moroder et al. [23]	2016	NR	65.4 (12.9)	25 (6)	4.2 (1)
von Engelhardt et al. [44]	2015	NS	NS	3.6 (2)	1.8 (1)
Teissier et al. [43]	2015	40 (24)	68 (12)	3.3 (3)	1.1 (1)
Kadum et al. [42]	2014	NR	NR	25 (4)	12.5 (2)
Ballas et al. [40]	2013	29 (8)	62 (12)	14.3 (8)	7.1 (4)

CS: Constant-Murley Score, rTSA: reverse total shoulder arthroplasty, SD: standard deviation, NR: not recorded, NS: not stratified.

**Table 3:** Incidence of postoperative complications in 637 patients who underwent stemless rTSA.

	Shoulders	Incidence (%)	Incidence of All Complications (%)
Instability and/or dislocation	16	2.5	19.5
Humeral implant displacement/malpositioning/migration	11	1.7	13.4
Superficial infection	1	0.2	1.2
Deep infection	4 *	0.6	4.9
Hematoma	4	0.6	4.9
Periprosthetic fracture (humerus)	12	1.9	14.6
Periprosthetic fracture (glenoid)	2	0.3	2.4
Periprosthetic fracture (unspecified)	2	0.3	2.4
Acromion fracture	6	0.9	7.3
Scapular spine fracture	1	0.2	1.2
Clavicle fracture	1	0.2	1.2
Glenosphere disassembly from baseplate	8	1.3	9.8
Dysesthesia in the hand	3	0.5	3.7
Postoperative stiffness	3	0.5	3.7
Subscapularis rupture	2	0.3	2.4
Symptomatic mesacromion	1	0.2	1.2
Chronic scapulothoracic conflict	1	0.2	1.2
Glenoid ossification	1	0.2	1.2
Glenoid and humeral loosening	1	0.2	1.2
Asymmetrical polyethylene	1	0.2	1.2
Incorrectly positioned humeral base plate	1	0.2	1.2
Overall complications	82	12.9	100.0

This table represents updated data from the study by Ajibade et al. [29]. * It is unclear whether deep infection occurred in the shoulder of a stemless or stemmed patient [41]. rTSA: reverse total shoulder arthroplasty.

**Table 4:** Comparative studies of stemmed and stemless rTSA.

Study	Year	Mean Age Year (SD)	Sex (Male %)	Mean BMI kg/m^2^ (SD)	Final Number of rTSA in Analysis	Implant	Mean Follow-Up Months (SD)	Main Findings
A’Court et al. [51]	2024	76.5 (6.3) vs. 64.3 (11.4)	53 vs. 40	29.2 (5.7) vs. 28.5 (4.5)	30 vs. 30	SMR (Lima Corporate)	31.3 (8.7) vs. 37.5 (14.0)	- No significant differences in PROMs, ROM - Four stemmed and seven stemless caused complications - Three stemless required revision
Moroder et al. [23]	2016	74.3 (4.6) vs. 75.6 (4.6)	29 vs. 29	NR	24 vs. 24	Delta XTEND (Depuy) TESS (Zimmer Biomet)	34.2 (10.5) vs. 35.2 (14.6)	- No significant differences in PROMs, ROM - Four stemmed and six stemless caused complications - Three stemmed and two stemless required revision
Kadum et al. [42]	2014	72 vs. 69	27 vs. 63	NR	15 vs. 16	TESS (Zimmer Biomet)	35 vs. 35	- Both groups improved in PROMs, ROM - Two stemless required revision

rTSA: reverse total shoulder arthroplasty, SD: standard deviation, BMI: body mass index, PROMs: patient-reported outcome measures, ROM: range of motion, NR: not reported. Each column represents stemmed vs. stemless.

**Table 5:** Randomized controlled trials comparing stemmed and stemless aTSA.

Study	Year	Mean Age Year (SD)	Sex (Male %)	Mean BMI kg/m^2^ (SD)	Final Number of aTSA in Analysis	Implant	Follow-Up	Main Findings
Romeo et al. [72]	2020	66.0 (median) vs. 66.0 (median)	73 vs. 69	31.8 (median) vs. 30.3 (median)	68 vs. 143	Univers II (Arthrex) Eclipse (Arthrex)	2 years	- No significant differences in PROMs - 3.8% of stemmed and 3.2% of stemless required revision
Wiater et al. [73]	2020	62.1 (9.6) vs. 63.1 (9.0)	65 vs. 67	30.1 (5.3) vs. 30.6 (5.8)	123 vs. 116	Comprehensive (Zimmer Biomet) Nano (Zimmer Biomet)	2 years	- No significant differences in PROMs, ROM - Nine stemmed and nine stemless caused failure
Uschok et al. [22]	2017	69 vs. 65	35 vs. 50	NR	18 vs. 15 (2 years) 15 vs. 14 (5 years)	Univers II (Arthrex) Eclipse (Arthrex)	2 and 5 years	- No significant differences in PROMs, ROM - One stemmed caused greater tuberosity fracture - 6.7% of stemmed and 7.1% of stemless required revision
Mariotti et al. [74]	2014	NS	NR	NR	10 vs. 9	Aequalis (Stryker Tornier)	2 years	- No significant differences in PROMs, ROM - No complications in either group

aTSA: anatomical total shoulder arthroplasty, SD: standard deviation, BMI: body mass index, PROMs: patient-reported outcome measures, ROM: range of motion, NR: not reported, NS: not stratified. Each column represents stemmed vs. stemless.

## Data Availability

Not applicable.

## Supplementary Materials

The following supporting information can be downloaded at: https://www.mdpi.com/article/10.3390/jcm13133813/s1, Table S1. Quality assessment of included studies using the JBI Critical Appraisal Tools for JBI Systematic Reviews.
