# Supplementary material for: Evolution of Stemless Reverse Shoulder Arthroplasty: Current Indications, Outcomes, and Future Prospects
Source: J Clin Med. 2024 Jun 28;13(13):3813. doi: 10.3390/jcm13133813 (PMC11242655; doi:10.3390/jcm13133813)
Supplement: Supplementary file 1 [file jcm-13-03813-s001.zip › jcm-3004200-supplementary.pdf]

Supplementary Table S1. Quality assessment of included studies using the JBI Critical Appraisal Tools for JBI Systematic Reviews.

	1. Was the sample frame appropriate to address the target population?	2. Were study participants sampled in an appropriate way?	3. Was the sample size adequate?	4. Were the study subjects and the setting described in detail?	5. Was the data analysis conducted with sufficient coverage of the identified sample?	6. Were valid methods used for the identificatio n of the condition?	7. Was the condition measured in a standard, reliable way for all participants ?	8. Was there appropriate statistical analysis?	9. Was the response rate adequate, and if not, was the low response rate managed appropriatel y?	Risk of bias	Overall appraisal
A'Court et al. [51]	Yes	Yes	NA	Yes	Yes	Yes	Yes	Yes	NA	Low	Include
Rosso et al. [50]	Yes	Yes	NA	Yes	Yes	Yes	Yes	Yes	NA	Low	Include
Nabergoj et al. [47]	Yes	Yes	NA	Yes	Yes	Yes	Yes	Yes	NA	Low	Include
Galhoum et al. [48]	Yes	Yes	NA	Unclear	Yes	Yes	Yes	Yes	NA	Moderate	Include
Schoch et al. [49]	Yes	Yes	NA	Yes	Yes	Yes	Yes	Yes	NA	Low	Include
Micheloni et al. [71]	Yes	Yes	NA	Yes	Yes	Yes	Yes	Yes	NA	Low	Include
Virani et al. [46]	Yes	Yes	NA	Unclear	Yes	Yes	Yes	Yes	NA	Moderate	Include
Beck et al. [39]	Yes	Yes	NA	Yes	Yes	Unclear	Yes	Yes	NA	Moderate	Include
Levy et al. [44]	Yes	Yes	NA	Yes	Yes	Yes	Yes	Yes	NA	Low	Include
Moroder et al. [23]	Yes	Yes	NA	Yes	Yes	Yes	Yes	Yes	NA	Low	Include
von Engelhardt et al. [42]	Yes	Yes	NA	Unclear	Yes	Yes	Yes	Yes	NA	Moderate	Include
Teissier et al. [41]	Yes	Yes	NA	Yes	Yes	Yes	Yes	Yes	NA	Low	Include
Kadum et al. [40]	Yes	Yes	NA	Yes	Yes	Yes	Yes	Yes	NA	Low	Include
Ballas et al. [39]	Yes	Yes	NA	Yes	Yes	Yes	Yes	Yes	NA	Low	Include

JBI, Joanna Briggs Institute; NA, not applicable
